# Assessment of Carotid Doppler Parameters to Evaluate Volume Status: A Prospective Observational Study

**DOI:** 10.1016/j.acepjo.2026.100533

**Published:** 2026-10-20

**Authors:** Haya Farhat, Gilbert Abou Dagher, Teisir Zaatari, Maha Makki, Hani Tamim, Ralph Bou Chebl

**Affiliations:** 1 Department of Emergency Medicine, American University of Beirut Medical Center, Beirut, Lebanon; 2 Department of Internal Medicine, American University of Beirut Medical Center, Beirut, Lebanon

**Keywords:** carotid, ultrasound, volume, status, VTI, flow time

## Abstract

**Objectives:**

Accurate assessment of volume status and fluid responsiveness is essential to guide fluid therapy, as unnecessary fluid administration may lead to fluid overload and adverse outcomes. Carotid Doppler ultrasound has emerged as a rapid, noninvasive alternative to more technically demanding echocardiographic measures. This study evaluated the utility of carotid Doppler-derived corrected flow time (FTc) and velocity-time integral (VTI), and their changes after passive leg raise (PLR), in assessing volume status and preload responsiveness.

**Methods:**

We conducted a prospective observational study during Ramadan at a tertiary academic medical center. A total of 202 participants were enrolled, including 103 fasting individuals assessed during presumed relative dehydration and after rehydration, and 99 nonfasting hydrated controls. Carotid Doppler measurements of FTc, VTI, and V_max_ were obtained before and after PLR. Diagnostic performance of percent changes in FTc (ΔFTc) and VTI (ΔVTI) was evaluated using receiver operating characteristic analysis.

**Results:**

Participants in the fasting group underwent a mean fasting duration of 12.4 ± 1.1 hours and consumed a mean of 920 ± 352 mL of fluid after breaking the fast. PLR induced significantly greater increases in FTc and VTI during presumed relative dehydration. A ≥10% increase in VTI was observed in 57.2% of presumed relative dehydrated participants compared with 26.3% of hydrated controls and 20.4% of rehydrated participants. Similarly, a ≥5% increase in FTc was observed in 47.6%, 17.2%, and 22.3% of participants, respectively. Receiver operating characteristic analysis demonstrated moderate discriminatory performance for presumed relative dehydration, with areas under the curve of 0.708 for ΔVTI and 0.715 for ΔFTc. Optimal cutoff values were 5.92% for ΔVTI and 5.05% for ΔFTc.

**Conclusion:**

Carotid Doppler-derived FTc and VTI are promising noninvasive markers of volume status and preload responsiveness. ΔVTI demonstrated greater sensitivity, whereas ΔFTc provided higher specificity, supporting the potential role of carotid Doppler ultrasound as a practical bedside tool for hemodynamic assessment.

**Bottom Line**

This study asked whether carotid Doppler ultrasound can detect changes in hydration status and response to increased blood flow using simple bedside measurements. Among 202 healthy volunteers, carotid Doppler-derived corrected flow time and velocity-time integral increased after passive leg raise, with larger changes during fasting-induced dehydration than after rehydration or in hydrated controls. Changes in carotid Doppler-derived corrected flow time and velocity-time integral showed moderate ability to distinguish dehydration from hydration, with areas under the curve of 0.715 and 0.708, respectively. These findings suggest that carotid ultrasound may provide a simple, noninvasive bedside approach for assessing hydration-related hemodynamic changes and fluid responsiveness.

## 1 Introduction

### 1.1 Background

Hypotension and shock are frequently encountered in critically ill patients and are associated with impaired tissue perfusion and reduced oxygen delivery.^1^, ^2^ If not promptly recognized and treated, they can lead to organ dysfunction, adverse outcomes, and death.^1^, ^2^ Management therefore requires rapid identification of the underlying pathophysiology while restoring adequate systemic perfusion and oxygen delivery.

Undifferentiated shock is a time-critical condition requiring rapid identification of the underlying pathophysiology to guide resuscitation. Point-of-care ultrasound (POCUS) has become an important component of the bedside assessment of shock, providing real-time information to help identify potentially reversible causes and guide management.^3^ The Rapid Ultrasound in Shock (RUSH) examination provides a structured approach to undifferentiated shock by integrating cardiac, pulmonary, abdominal, and vascular findings to distinguish the major shock phenotypes.^3^ Hypovolemic shock is characterized by findings suggestive of reduced intravascular volume, whereas cardiogenic shock may be identified by impaired cardiac function and evidence of elevated filling pressures. Obstructive shock can be suggested by findings such as right ventricular strain, pericardial effusion with tamponade physiology, or pneumothorax, whereas distributive shock may demonstrate hyperdynamic or, in advanced cases, depressed cardiac function with variable intravascular volume findings.^3^ By integrating these findings through the “pump, tank, and pipes” framework, RUSH can rapidly narrow the differential diagnosis and identify conditions requiring immediate targeted intervention.^3^

### 1.2 Importance

Although POCUS can help identify the cause of shock, determining whether a patient will benefit from additional intravenous fluid remains challenging. Intravenous fluids are commonly used during initial resuscitation to improve systemic perfusion and oxygen delivery,^2^, ^4^ but further administration requires careful assessment.^4^, ^5^, ^6^, ^7^ Fluid status describes a patient’s intravascular volume state, whereas fluid responsiveness describes the cardiovascular response to increased preload and is commonly defined as a 10% to 15% increase in stroke volume after a fluid challenge.^6^, ^7^ Distinguishing responders from nonresponders is important because unnecessary fluid administration may contribute to fluid overload, morbidity, and adverse outcomes.^4^, ^5^

Various methods have been proposed to assess volume status and predict fluid responsiveness, including central venous pressure, inferior vena cava (IVC) size and respiratory variation, and other ultrasound-derived indices.^6^, ^8^, ^9^ Although IVC ultrasound is commonly used to assess intravascular volume, static measures alone may not reliably determine whether cardiac output will increase with additional preload.^6^, ^7^, ^8^

Dynamic ultrasound-derived measures may provide additional information. Left ventricular outflow tract velocity-time integral (LVOT VTI) has been used to assess changes in stroke volume and predict fluid responsiveness, particularly in response to passive leg raise (PLR).^8^, ^9^, ^10^ However, accurate LVOT VTI acquisition requires appropriate echocardiographic windows, precise probe positioning, and optimal Doppler alignment. Patient factors, such as body habitus, may further limit transthoracic windows, reducing feasibility in time-sensitive or technically challenging settings.

Carotid artery ultrasound has, therefore, emerged as a potential noninvasive alternative for assessing hemodynamic changes and fluid responsiveness.^11^, ^12^, ^13^, ^14^ The common carotid artery is superficial and readily accessible, making Doppler measurements potentially easier to obtain than LVOT measurements. Corrected flow time (FTc) and carotid VTI provide objective, quantitative measurements that can be assessed before and after a dynamic preload challenge. FTc reflects left ventricular systolic ejection duration corrected for heart rate, whereas carotid VTI represents the distance traveled by blood during systole and may serve as a surrogate for changes in stroke volume. Previous studies have demonstrated changes in these parameters with alterations in preload and explored their utility in assessing volume status and fluid responsiveness.^11^, ^12^, ^13^, ^14^, ^15^, ^16^, ^17^, ^18^, ^19^

### 1.3 Goals of This Investigation

In this study, we sought to evaluate carotid Doppler-derived FTc and VTI as noninvasive markers of volume status and preload responsiveness. Specifically, we aimed to characterize changes in FTc and VTI following PLR across presumed relative dehydrated, rehydrated, and hydrated states; assess the ability of PLR-induced relative changes in FTc (ΔFTc) and VTI (ΔVTI) to discriminate between dehydrated and hydrated states; and determine optimal cutoff values. We hypothesized that PLR-induced changes in FTc and VTI would be greater during fasting-induced dehydration than after rehydration or in nonfasting hydrated controls.

## 2 Methods

### 2.1 Study Design

We conducted a prospective observational study during the month of Ramadan to evaluate carotid Doppler-derived parameters across different hydration states and their responses to PLR. The study protocol was approved by the Institutional Review Board (IRB #BIO-2026-017), and all participants provided informed consent before enrollment.

### 2.2 Setting

The study was conducted in the emergency department of the American University of Beirut Medical Center (AUBMC), a tertiary academic medical center in Beirut, Lebanon. Ultrasound examinations were performed in a designated room within the emergency department using a standardized ultrasound acquisition protocol.

### 2.3 Selection of Participants

Participants were voluntarily recruited from emergency department personnel, including resident physicians, nurses, attending physicians, and administrative staff. Participants observing a prolonged fast of approximately 12 hours were enrolled in the fasting study group. Nonfasting participants were enrolled separately as hydrated controls.

Participants in the fasting group underwent an initial ultrasound assessment while fasting and a second assessment approximately 4 hours after breaking the fast. Participants reported their fasting duration and the amount of fluid consumed after breaking the fast. Nonfasting participants underwent ultrasound assessment while normally hydrated and served as the hydrated control group.

### 2.4 Sample Size

Sample size estimation was based on findings from a previously published study by Shokoohi et al,^20^ which evaluated carotid corrected flow time (FTc) in participants undergoing prolonged fasting and subsequent rehydration. The study reported mean FTc values of 312 ± 22 ms during the fasting state and 345 ± 25 ms after rehydration, corresponding to a difference of approximately 33 ms.^20^ Based on this difference and the reported standard deviations, an estimated sample size of 9 participants per group would provide 80% power, whereas 12 participants per group would provide 90% power. Given the relatively small sample size generated by this calculation, we elected to enroll a larger sample based on study feasibility and participant availability, ultimately enrolling 103 participants in the fasting study group. In our cohort, FTc increased from 347.15 ± 37.76 ms before PLR to 365.16 ± 43.42 ms after PLR, with a standard deviation of the paired difference of 20.25 ms.

### 2.5 Exposures

The exposure of interest was hydration state. Three physiologic states were evaluated: presumed relative dehydration, postrehydration, and nonfasting normal hydration. Measurements obtained after approximately 12 hours of fasting were considered to represent the dehydrated state. Repeat measurements obtained approximately 4 hours after breaking the fast were considered to represent the rehydrated state. Measurements obtained from nonfasting control participants were considered to represent the hydrated state. For simplicity and ease of read, these 3 states are hereafter referred to as the presumed dehydrated, presumed rehydrated, and presumed hydrated states, respectively.

Because fluid intake during the 4 hours after breaking the fast might not have been sufficient to completely restore hydration, a separate nonfasting control group was included to represent a normally hydrated state. The amount of fluid consumed after breaking the fast was recorded for each fasting participant.

### 2.6 Ultrasound Measurements

Ultrasound examinations were performed using a GE LOGIQ e ultrasound system (General Electric) with a linear-array transducer operating at a frequency range of 4.2 to13 MHz. The factory carotid preset was used for image acquisition.

An ultrasound-trained emergency medicine faculty member and an emergency ultrasound fellow performed the ultrasound examinations independently. The fellow received extensive training on carotid scanning prior to the start of the study. Each participant was scanned by a single operator. The ultrasound-trained emergency medicine faculty member subsequently reviewed all ultrasound examinations to ensure image quality and measurement accuracy. Faculty review consisted only of image quality assessment.

Doppler measurements of the common carotid artery were obtained in the longitudinal axis immediately proximal to the carotid bulb. Participants were positioned in a semi-Fowler position with the head rotated away from the side of measurement. Angle correction was applied and maintained at ≤60° relative to the direction of blood flow.

At each assessment, carotid flow time, VTI, and maximum velocity (V_max_) were measured before and after PLR. Flow time was defined as the interval from the onset of systole to the dicrotic notch within each cardiac cycle. Corrected flow time (FTc) was calculated using Wodey formula:

FTc=FT+1.29×(HR−60).

VTI was calculated by tracing the spectral Doppler waveform over 1 cardiac cycle, representing the distance traveled by blood during systole.

Heart rate was recorded at the time of each measurement. Demographic and clinical characteristics, including age, sex, smoking status, and comorbidities, were also recorded.

Ultrasound operators were not blinded to participants’ fasting status. Blinding was not feasible because fasting status and fasting duration were obtained directly from participants as part of the study protocol. In addition, operators were familiar with the participants and their religious and cultural backgrounds, which could provide contextual information regarding fasting status.

### 2.7 Outcomes

The primary outcome was the diagnostic performance of the relative percent changes in carotid FTc (ΔFTc) and VTI (ΔVTI) after PLR for discriminating between presumed dehydrated and presumed hydrated states. This outcome was selected to evaluate whether dynamic changes in carotid Doppler parameters after preload augmentation could distinguish relative volume depletion from normal hydration.

Secondary outcomes included differences in baseline Doppler-derived parameters across hydration states; within-state changes in FTc, VTI, and V_max_ following PLR; and the distribution of percent changes in these parameters across presumed dehydrated, presumed rehydrated, and presumed hydrated states. The relationship between the amount of fluid consumed after breaking the fast and PLR-induced changes in FTc and VTI was also evaluated. Optimal cutoff values for ΔFTc and ΔVTI for distinguishing presumed dehydrated from presumed hydrated states were determined.

### 2.8 Data Analysis

The Statistical Package for Social Sciences, version 31.0 was used for data entry, management, and analyses. Descriptive statistics were used to summarize participant characteristics and Doppler-derived measurements. Continuous variables were reported as mean ± standard deviation, and categoric variables—as counts and percentages. The association between hydrated control, fasting group, and other categoric variables was assessed using the chi-square test, whereas Student t-test was used to assess continuous variables. A paired t-test was used to assess within-group changes before and after PLR for each hydration state. Diagnostic accuracy was evaluated using sensitivity, specificity, positive predictive value, and negative predictive value. Receiver operating characteristic (ROC) curve analysis was performed to assess the discriminatory ability of ΔFTc and ΔVTI for identifying dehydration, with the area under the curve (AUC) used to quantify overall performance. Optimal cutoff values were selected based on the best balance between sensitivity and specificity. *P* value < .05 was used to indicate statistical significance.

## 3 Results

A total of 202 participants were enrolled, including 99 participants in the presumed hydrated control group and 103 participants in the fasting study group. Participants in the fasting group underwent assessment during fasting (presumed dehydrated state) and again after breaking the fast (presumed rehydrated state).

Baseline demographic and clinical characteristics are summarized descriptively in Table 1. The groups had similar mean ages (35.32 ± 10.36 vs 34.06 ± 8.39 years) and mean heart rates (74.63 ± 8.91 vs 73.18 ± 8.21 beats/min). Smoking prevalence was also similar between the presumed hydrated control and fasting groups (40.4% vs 41.5%). Differences were observed in the distribution of some baseline characteristics, including sex (65.7% vs 57.3% male), hypertension (11.1% vs 7.8%), diabetes mellitus (10.1% vs 2.9%), and dyslipidemia (9.1% vs 5.8%). These differences were considered descriptively and were not judged to represent clinically meaningful baseline imbalances likely to substantially influence the study comparisons.

**Table 1:** Baseline demographic and clinical characteristics of the study population.

Characteristic	Presumed Hydrated Control (n = 99)	Fasting Study Group (n = 103)
Sex, n (%)		
Male	65 (65.7)	59 (57.3)
Female	34 (34.3)	44 (42.7)
Age, y, mean ± SD	35.32 ± 10.36	34.06 ± 8.39
Smoking, n (%)	40 (40.4)	43 (41.5)
Hypertension, n (%)	11 (11.1)	8 (7.8)
Diabetes mellitus, n (%)	10 (10.1)	3 (2.9)
Dyslipidemia, n (%)	9 (9.1)	6 (5.8)
Coronary artery disease, n (%)	1 (1.0)	2 (1.9)
Heart rate, beats/min, mean ± SD	74.63 ± 8.91	73.18 ± 8.21
Fasting duration, hours, mean ± SD	-	12.36 ± 1.09
Interval between ultrasound measurements, hours, mean ± SD	-	3.81 ± 0.58
Fluid intake after breaking the fast, mL, mean ± SD	-	920.39 ± 351.78

Characteristics of the hydrated control (n = 99) and fasting study (n = 103) groups. Fasting-specific variables were applicable only to the fasting group. Data are presented as mean ± SD or n (%), as appropriate.

Among fasting participants, the mean fasting duration was 12.36 ± 1.09 hours. The mean interval between ultrasound assessments was 3.81 ± 0.58 hours, and participants consumed a mean of 920.39 ± 351.78 mL of fluid after breaking the fast.

### 3.1 Mean Doppler-Derived Parameters

Table 2 presents the mean Doppler-derived hemodynamic parameters across the three hydration states. Mean FTc was 347.15 ± 37.76 ms in the fasting group, 354.86 ± 39.29 ms after rehydration, and 355.35 ± 23.73 ms in the presumed hydrated group. Mean VTI was 21.44 ± 3.38 in the fasting group, 21.83 ± 3.12 after rehydration, and 22.05 ± 4.57 in the presumed hydrated group. Mean V_max_ was 106.89 ± 15.52 cm/s in the fasting group, 113.00 ± 16.36 cm/s after rehydration, and 123.09 ± 29.19 cm/s in the presumed hydrated group.

**Table 2:** Changes in Doppler-derived hemodynamic parameters after passive leg raise across hydration states.

Parameter	Presumed Dehydrated State (n = 103)			Presumed Rehydrated State (n = 103)			Presumed Hydrated Group (n = 99)		
	Pre-PLR	Post-PLR	*P* value	Pre-PLR	Post-PLR	*P* value	Pre-PLR	Post-PLR	*P* value
FTc, ms	347.15 ± 37.76	365.16 ± 43.42	<.001	354.86 ± 39.29	364.20 ± 35.02	<.001	355.35 ± 23.73	366.48 ± 28.59	<.001
VTI	21.44 ± 3.38	23.99 ± 3.60	<.001	21.83 ± 3.12	23.02 ± 3.54	<.001	22.05 ± 4.57	23.26 ± 4.81	<.001
V_max_, cm/s	106.89 ± 15.52	113.50 ± 16.17	<.001	113.00 ± 16.36	116.73 ± 16.88	<.001	123.09 ± 29.19	129.82 ± 30.50	.002

Values are presented as mean ± SD. P values represent within-state comparisons of pre- versus post-PLR measurements.

FTc, corrected flow time; PLR, passive leg raise; V_max_, maximum velocity; VTI, velocity-time integral.

### 3.2 Changes in Doppler-Derived Parameters After PLR

The changes in Doppler-derived hemodynamic parameters following PLR across the three hydration states are also presented in Table 2. In the presumed dehydrated fasting state, FTc increased from 347.15 ± 37.76 to 365.16 ± 43.42 ms, VTI increased from 21.44 ± 3.38 to 23.99 ± 3.60, and V_max_ increased from 106.89 ± 15.52 to 113.50 ± 16.17 cm/s, (all *P* < .001). After breaking their fast, in the presumed rehydrated state, FTc increased from 354.86 ± 39.29 to 364.20 ± 35.02 ms, VTI increased from 21.83 ± 3.12 to 23.02 ± 3.54, and V_max_ increased from 113.00 ± 16.36 to 116.73 ± 16.88 cm/s, (all *P* < .001). In the nonfasting presumed hydrated group, FTc increased from 355.35 ± 23.73 to 366.48 ± 28.59 ms (*P* < .001), VTI increased from 22.05 ± 4.57 to 23.26 ± 4.81 (*P* < .001), and V_max_ increased from 123.09 ± 29.19 to 129.82 ± 30.50 cm/s (*P* = .002).

### 3.3 Distribution of Percent Change After PLR

As shown in Table 3, when looking at the percent change in parameters after the PLR maneuver, 57.2% of participants in the presumed dehydrated state had a ≥10% increase in VTI after PLR, compared with 26.3% in the presumed hydrated group and 20.4% after rehydration. Similarly, 12.6% of presumed dehydrated patients had a ≥10% increase in FTc after PLR, compared with 5.0% in the presumed hydrated group and 9.7% after rehydration. For V_max_, a ≥10% increase was observed in 27.2% of presumed dehydrated participants, compared with 23.2% in the presumed hydrated group and 20.4% after rehydration.

**Table 3:** Distribution of percent changes in Doppler-derived parameters after passive leg raise across hydration states.

Percent Change	Presumed Dehydrated State (n = 103)	Presumed Rehydrated State (n = 103)	Presumed Hydrated Group (n = 99)
**VTI: change after PLR, n (%)**			
<5%	19 (18.4)	52 (50.5)	53 (53.5)
5-10%	25 (24.3)	30 (29.1)	20 (20.2)
10-15%	26 (25.2)	9 (8.7)	11 (11.1)
>15%	33 (32.0)	12 (11.7)	15 (15.2)
**FTc: change after PLR, n (%)**			
<5%	54 (52.4)	80 (77.7)	82 (82.8)
5-10%	36 (35.0)	13 (12.6)	12 (12.1)
10-15%	10 (9.7)	7 (6.8)	4 (4.0)
>15%	3 (2.9)	3 (2.9)	1 (1.0)
**V_max_: change after PLR, n (%)**			
<5%	53 (51.5)	75 (72.8)	56 (56.6)
5%-10%	22 (21.4)	7 (6.8)	20 (20.2)
10%-15%	13 (12.6)	8 (7.8)	12 (12.1)
>15%	15 (14.6)	13 (12.6)	11 (11.1)

Values represent n (%) of participants within each hydration state according to the percent change from pre- to post-PLR measurements.

FTc, corrected flow time**;** PLR, passive leg raise; V_max_, maximum velocity; VTI, velocity-time integral.

### 3.4 Effect of Postfast Fluid Intake on Doppler Responsiveness

To explore the relationship between postfast fluid intake and preload responsiveness, fasting participants were stratified according to whether they consumed less than 1 L or at least 1 L of fluid after breaking the fast.As shown in Table 4, participants who consumed less than 1 L demonstrated numerically greater PLR-induced Doppler responses compared with those who consumed at least 1 L. A ≥10% increase in VTI following PLR was observed in 61.4% of participants who consumed less than 1 L compared with 54.2% of those who consumed at least 1 L (*P* = .47). Similarly, a ≥5% increase in FTc was observed in 56.8% of participants who consumed less than 1 L compared with 40.7% of those who consumed at least 1 L (*P* = .11).

**Table 4:** Association between postfluid intake and PLR-induced changes in carotid VTI and FTc.

PLR Response	<1 L (n = 44)	≥1 L (n = 59)	*P* value
**ΔVTI response, n (%)**			.47
≥10%	27 (61.4)	32 (54.2)	
<10%	17 (38.6)	27 (45.8)	
**ΔFTc response, n (%)**			.11
≥5%	25 (56.8)	24 (40.7)	
<5%	19 (43.2)	35 (59.3)	

Participants in the fasting group were categorized based on fluid intake after breaking the fast (<1 L vs ≥1 L). Values are presented as n (%). P values compare response categories between the 2 fluid-intake groups.

ΔFTc, relative percent change in corrected flow time after PLR; ΔVTI, relative percent change in velocity-time integral after PLR; Ftc, carotid Doppler-derived corrected flow time; PLR, passive leg raise.

### 3.5 Diagnostic Performance of Doppler-Derived Percent Change

For discriminating the presumed dehydrated from the presumed hydrated state, ΔFTc > 5% demonstrated high specificity (92.5%, 95% CI 81.8-97.9) but low sensitivity (28.3%, 95% CI 16.0-43.5), with a positive predictive value of 76.5% (95% CI 53.2-90.3) and a negative predictive value (NPV) of 59.8% (95% CI 54.9-64.4). At the ≥10% ΔFTc cutoff, specificity remained high at 94.2% (95% CI 86.9-98.1), while sensitivity was 0.0% (95% CI 0.0 to 24.7), with an NPV of 86.2% (95% CI 85.5-86.8). For ΔVTI ≥10%, specificity and positive predictive value were both 100.0% (95% CI 92.0-100.0 and 86.8-100.0, respectively), while sensitivity was 47.2% (95% CI 33.6-61.2) and NPV was 60.3% (95% CI 54.2 to 66.1).

As illustrated in Figure and summarized in Table 5, ROC analysis showed comparable discriminatory performance for ΔVTI and ΔFTc, with AUCs of 0.708 (95% CI 0.635-0.780) and 0.715 (95% CI 0.643-0.786), respectively. The optimal cutoff to differentiate a presumed dehydrated state from a presumed hydrated was 5.92% for ΔVTI, yielding a sensitivity of 0.79 and specificity of 0.60, and 5.05% for ΔFTc, yielding a sensitivity of 0.47 and specificity of 0.75.

**Figure:**
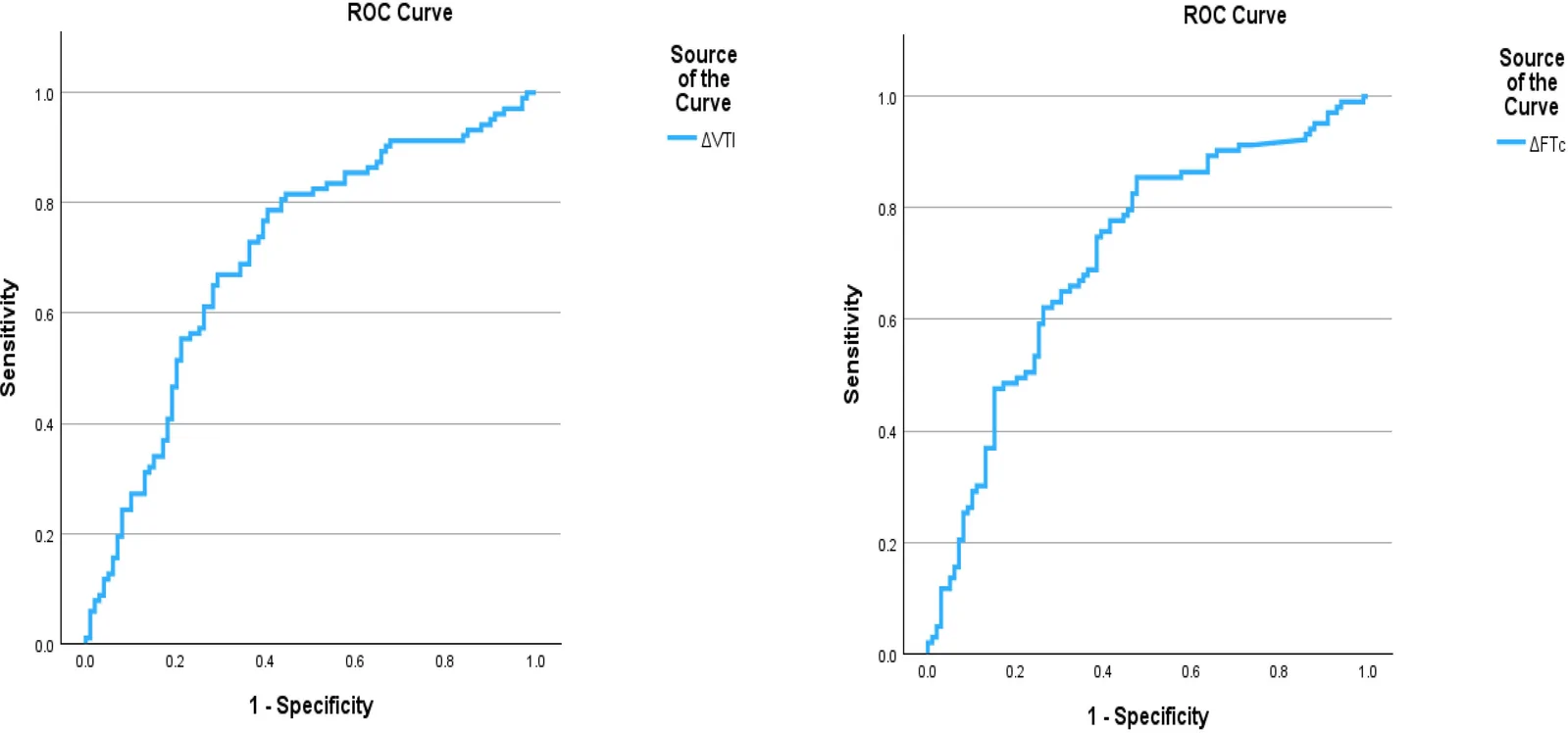
Receiver operating characteristic curves (ROC) for ΔVTI and ΔFTc. ROC curves for discriminating dehydrated from hydrated states.

**Table 5:** Diagnostic performance of PLR-induced changes in carotid VTI and FTC for discriminating dehydrated from hydrated states.

Variable	AUC	95% CI	Optimal Cutoff	Sensitivity	Specificity
ΔVTI	0.708	0.635-0.780	5.92	0.79	0.60
ΔFTc	0.715	0.643-0.786	5.05	0.47	0.75

ROC analysis was used to evaluate ΔVTI and ΔFTc for distinguishing the fasting dehydrated state from the nonfasting hydrated state. Optimal cutoff values represent percent change after PLR.

ΔFTc, relative percent change in corrected flow time after PLR; ΔVTI, relative percent change in velocity-time integral after PLR; AUC, area under the curve; PLR, passive leg raise; ROC, receiver operating characteristic.

## 4 Limitations

Several limitations should be acknowledged. First, the study population consisted of healthy volunteers rather than critically ill patients, which may limit generalizability to emergency department or intensive care populations with shock. Second, hydration status was defined according to fasting and rehydration status rather than invasive hemodynamic monitoring or laboratory biomarkers. Third, although PLR was used as a reversible preload challenge, fluid responsiveness was not confirmed using invasive cardiac output monitoring. Fourth, carotid Doppler measurements may be affected by operator technique despite standardized training and subsequent review by an ultrasound fellowship-trained attending physician. Additionally, BMI was not collected, precluding assessment of the potential influence of body habitus on ultrasound feasibility and image quality. Fifth, ultrasound operators were not blinded to participants’ fasting or hydration status, which may have introduced measurement or observer bias. Blinding was not feasible because fasting status and fasting duration were obtained directly from participants as part of the study protocol. Finally, vascular tone, sympathetic activation, and individual variability in fasting physiology may influence carotid Doppler parameters independently of intravascular volume.

## 5 Discussion

In this prospective observational study, carotid Doppler-derived FTc, and VTI showed significant changes after PLR, with larger responses observed during fasting-induced volume depletion than after rehydration or in nonfasting hydrated controls. These findings suggest that carotid Doppler parameters may provide a practical, noninvasive method for detecting hemodynamic changes associated with relative intravascular volume depletion. Although Ramadan fasting has previously been used as a physiologic model for evaluating volume-related changes, our study reinforces these observations while extending the assessment to include both carotid FTc and VTI and their dynamic responses to PLR. An additional strength of our design was the inclusion of a separate nonfasting hydrated control group. Because fluid intake during the 4 hours after breaking the fast may not have been sufficient to fully restore hydration, the control group provided a reference for normal hydration rather than assuming that postfast rehydration represented complete restoration of volume status. This design allowed carotid Doppler responses to be evaluated across 3 physiologic states—presumed dehydrated, presumed rehydrated, and presumed hydrated—and enabled assessment of both FTc and VTI within the same study population.

Corrected flow time reflects the duration of left ventricular ejection and is influenced by preload, afterload, contractility, and heart rate.^15^, ^16^, ^18^, ^19^ Because systolic ejection time shortens as heart rate increases, correction formulas such as Wodey formula are used to reduce heart rate-related variability and improve comparability between measurements.^15^ We selected Wodey formula because it provides a direct heart rate correction, has demonstrated strong concordance with changes in stroke volume, and allows FTc to be calculated from flow time and recorded heart rate without requiring a separate measurement of cardiac cycle time.^21^ In hypovolemic states, reduced preload may shorten systolic ejection time, whereas preload augmentation through PLR or fluid administration may increase FTc. Previous studies have demonstrated that changes in carotid FTc after PLR or volume expansion correlate with fluid responsiveness in critically ill, perioperative, and dialysis populations.^15^, ^16^, ^18^, ^22^ Barjaktarevic et al^15^ demonstrated that increases in carotid FTc after PLR correlated with fluid responsiveness in patients with undifferentiated shock, while Antiperovitch et al^18^ observed dynamic changes in carotid systolic flow time during dialysis-associated volume shifts. Similarly, Jalil et al^19^ reported that PLR-induced changes in carotid flow time correlated with changes in stroke volume, supporting the physiologic relationship between FTc and preload-dependent cardiovascular responses.

Our results demonstrated that FTc increased after PLR, with the largest changes occurring in presumed dehydrated participants. Approximately 35% of dehydrated individuals exhibited a 5% to 10% increase in FTc compared with 12.6% of presumed rehydrated participants and 12.1% of presumed hydrated controls. This finding suggests that the endogenous volume challenge generated by PLR increased systolic ejection time most prominently in individuals with relative volume depletion. Mean FTc was lower in fasting participants (347.15 ± 37.76 ms) than in both rehydrated participants (354.86 ± 39.29 ms) and hydrated controls (355.35 ± 23.73 ms). However, substantial overlap between groups suggests that absolute FTc values alone may be less useful for distinguishing hydration states. Individual differences in vascular tone, autonomic regulation, and cardiac function may contribute to this variability. Collectively, these findings suggest that ΔFTc may be a more useful dynamic marker of hemodynamic responsiveness to preload augmentation than a standalone indicator of volume depletion.

Carotid VTI represents the distance traveled by blood during systole and serves as a surrogate marker of stroke volume. Because carotid Doppler imaging is technically simpler and more rapidly obtainable than LVOT measurements, it has attracted growing interest as a bedside hemodynamic tool.^11^, ^17^ In our study, VTI increased significantly after PLR across all hydration states, with the largest response observed during fasting-induced dehydration. More than half of participants in the presumed dehydrated state demonstrated a ≥10% increase in VTI after PLR, compared with substantially smaller proportions in the presumed hydrated and presumed rehydrated groups. These findings suggest that carotid VTI may be particularly sensitive to preload augmentation in individuals with relative volume depletion.^17^

The diagnostic performance of both ΔVTI and ΔFTc was moderate, with AUCs of 0.708 and 0.715, respectively. These findings are generally consistent with previous studies evaluating carotid Doppler parameters as markers of preload responsiveness. Barjaktarevic et al^15^ reported an AUC of 0.88 for PLR-induced changes in FTc among critically ill patients, whereas Dai et al^17^ found an AUC of 0.821 for carotid VTI in predicting fluid responsiveness. In contrast, Bilgili et al^22^ reported lower discriminatory performance for FTc (AUC 0.58) in a geriatric surgical population, highlighting the variability of carotid Doppler performance across clinical settings. Compared with these studies, our cohort showed more modest diagnostic accuracy. This difference likely reflects the unique characteristics of our study population, which consisted of healthy fasting volunteers rather than patients with acute circulatory failure. Consequently, the degree of hemodynamic disturbance and volume depletion was likely less pronounced than that observed in critically ill populations. Nevertheless, the observed AUC values suggest that both ΔVTI and ΔFTc may provide clinically useful information regarding hemodynamic responses to preload augmentation and warrant further investigation in patient populations where fluid assessment is clinically relevant.

The present findings also reinforce the importance of dynamic assessment. Static measurements of volume status, such as isolated vessel diameter or single baseline Doppler values, may not reliably predict whether a patient will respond to additional preload. PLR provides a reversible endogenous volume challenge by transiently increasing venous return without administering intravenous fluid. Monnet and Teboul^8^ emphasized that PLR is most useful when paired with real-time measurement of cardiac output or a reliable surrogate of stroke volume. In this study, carotid Doppler measurements obtained before and after PLR allowed assessment of dynamic changes in FTc and VTI, which likely explains their ability to reflect hydration-related hemodynamic differences more effectively than baseline values alone.

Participants who consumed less than one liter of fluid after breaking their fast tended to demonstrate greater PLR-induced increases in both VTI and FTc compared with those who consumed one liter or more. Although these differences did not reach statistical significance, the observed trend supports the physiologic relationship between hydration status and carotid Doppler-derived dynamic parameters and suggests that lower fluid intake may be associated with persistent preload responsiveness despite rehydration.

In conclusion, this study supports the potential role of carotid Doppler-derived FTc and VTI as noninvasive markers of volume status and preload responsiveness. Both parameters increased significantly after PLR, with greater changes observed during fasting-induced dehydration. ΔVTI appeared more sensitive for detecting preload-responsive physiology, making it a better screening marker, whereas ΔFTc demonstrated higher specificity, making it a better confirmatory marker. These findings are consistent with prior studies evaluating carotid Doppler ultrasound for fluid responsiveness and suggest that carotid VTI and FTc may serve as practical bedside tools for volume assessment.^15^, ^16^, ^17^, ^18^, ^19^, ^20^, ^22^ Further studies in acutely ill and hemodynamically unstable patients are warranted to validate these findings and determine clinically applicable cutoff values.

## Author Contributions

HF, GAD, TZ, MM, HT, and RBC contributed to the study conception and design. Data collection was performed by HF and members of the study team. Statistical analysis was performed by MM and HT. HF drafted the manuscript. All authors contributed to manuscript revision, reviewed the final version, and approved the manuscript for submission.

## Funding and Support

By *JACEP Open* policy, all authors are required to disclose any and all commercial, financial, and other relationships in any way related to the subject of this article as per ICMJE conflict of interest guidelines (see www.icmje.org). This study did not receive any funding.

## Conflicts of Interest

The authors declare that they have no known competing financial interests or personal relationships that could have appeared to influence the work reported in this manuscript.
